# Iron doped gold cluster nanomagnets: *ab initio* determination of barriers for demagnetization†

**DOI:** 10.1039/c8na00359a

**Published:** 2019-02-12

**Authors:** Christopher Ehlert, Ian P. Hamilton

**Affiliations:** Department of Chemistry and Biochemistry, Wilfrid Laurier University 75 University Ave W Waterloo ON N2L3C5 Canada, cehlert@wlu.ca ihamilton@wlu.ca

## Abstract

Magnetic properties of small- and nano-sized iron doped gold clusters are calculated at the level of second order multireference perturbation theory. We first assess the methodology for small Au_6_Fe and Au_7_Fe clusters, which are representative of even and odd electron count systems. We find that larger active spaces are needed for the odd electron count system, Au_7_Fe, which exhibits isotropic magnetization behaviour. On the other hand, the even electron count system, Au_6_Fe, exhibits strong axial magnetic anisotropy. We then apply this methodology to the tetrahedral and truncated pyramidal nano-sized Au_19_Fe (with *S* = 3/2) and Au_18_Fe (with *S* = 2) clusters. We find that face substitutions result in the most stable structures, followed by edge and corner substitutions. However, for Au_18_Fe, corner substitution results in strong magnetic anisotropy and a large barrier for demagnetization while face substitution does not. Thus, although corner and face substituted Au_18_Fe have the same spin, only corner substituted Au_18_Fe can act as a single nanoparticle magnet.

## Abstract 2

Magnetic properties of small- and nano-sized iron doped gold clusters are calculated at the level of second order multireference perturbation theory.

## Graphical abstract

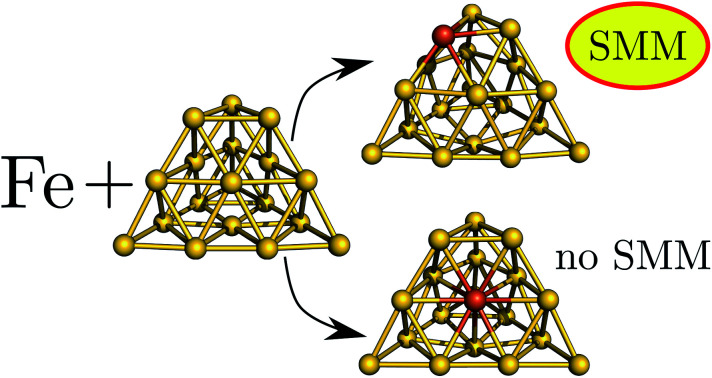

## 1 Introduction

Small- to nano-sized gold clusters show considerably different properties compared to bulk gold.^1^ Remarkable structural variations, such as two dimensional flakes, and three dimensional compact, cage and tube structures have been found.^2–7^ Gold clusters usually exhibit small (*S* = 0, 1/2) spin quantum numbers, leading to closed shell or doublet electronic ground states but doping them with a transition metal atom can lead to open-shell clusters with significantly higher spin-quantum numbers.^8–14^ For example, Tam *et al.*^12^ investigated the structure and stability of transition metal doped golden pyramids Au_19_M (M = Cr, Mn, Fe) at the level of generalized gradient density functional theory (DFT). All systems show higher ground state spin quantum numbers, while the structural modifications are minor. Similar findings have also been reported by Wang *et al.*^14^ and Yang *et al.*^10^ for golden cages containing a centrally trapped transition metal atom (M@Au_16_ and M@Au_24_). Doping a gold cluster with a transition metal atom therefore provides an opportunity to combine the structural diversity of gold clusters with the unique properties of high-spin systems. We note that experimental evidence of Au_*m*_Fe_*n*_ (*m* = 1–35, *n* = 1–3) structures has been found by Mawale *et al.* using mass spectrometry after laser desorption ionization of a gold-iron nanoflower.^15^

Single molecule magnets (SMMs) can be seen as a special class of open-shell systems with distinct properties. For example, the first investigated SMM, the dodecanuclear manganese acetate cluster [Mn_12_O_12_(CH_3_COO)_16_(H_2_O)_4_]·2CH_3_COOH·4H_2_O^16–18^ exhibits a ground state spin quantum number of *S* = 10 and can be magnetized by an external magnetic field. Once the field is switched off, the system relaxes *via* various channels back to the initial non-magnetized state. Characteristic for SMMs is a high relaxation barrier, which significantly slows the demagnetization process at low temperatures. The higher the demagnetization barrier, the longer the magnetization can be retained, which increases the potential for information storage and other applications. The demagnetization barrier is proportional to |*D*|*S*^2^,^19^ where *S* is the spin quantum number and *D* is the axial zero-field splitting (ZFS) parameter. Together with the rhombicity parameter, *E*/*D*, these enter the field-free part of the spin-Hamiltonian. If complemented with the field-dependent part, the spin-Hamiltonian can be written as:^20,21^1
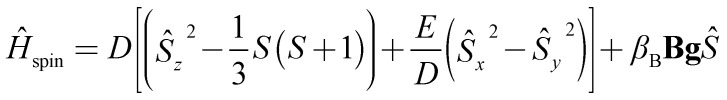
where *Ŝ*_*k*_ are spin operators while *β*_B_, **B**, and **g** are the Bohr magneton, the external magnetic field, and the **g**-tensor, respectively. The latter quantity, which describes the interaction with the external magnetic field, can be written as **g** = 1*g*_e_ + Δ**g** where *g*_e_ is the *g*-value for the free electron and Δ**g** are the shifts. The spin-Hamiltonian describes the splitting of the (2*S* + 1) manifold of the electronic ground state, which results from spin–orbit and spin–spin interactions.^22^ As outlined by Atanasov *et al.*,^20^ besides large *S*, SMM candidates should have large *D* (with negative sign) ensuring that the barrier for demagnetization is large. Also, they should have a small rhombicity parameter ensuring that “tunneling” through the demagnetization barrier is slow. As such, to assess the capability of an open-shell system to be used as a potential SMM, it is necessary to calculate the ZFS parameters. Finally, we note that the two requirements for SMM characteristics result in a large axial magnetic anisotropy of the system (which can be checked by inspecting eqn (1), for *D* ≪ 0, *E*/*D* = 0).

The central aim of this paper is the *ab initio* determination of the ZFS parameters (and, as such, magnetic anisotropies) for iron doped gold clusters. Several studies have investigated transition metal doped gold clusters using DFT.^8–14^ These studies focused on predicting stable structures and their spin quantum numbers. On the other hand, several studies examined magnetic properties of transition metal complexes, *i.e.*, a transition metal ion surrounded by a primarily organic framework, with wave function based, multireference perturbation theory methods.^23–25^ In addition, Aravena *et al.* studied transition metal ions in an inorganic polyoxometalate environment.^26^ To our present knowledge, our study is the first to report calculations of magnetic properties of transition metal doped gold clusters at the level of multireference perturbation theory. In particular, we use an approach based on SA-CASSCF^27^/NEVPT2 (ref. 28–31) (state averaged complete active space self-consistent field/second order n-electron valence perturbation theory) as implemented in the ORCA program^32^ (for a detailed description, see the ESI†). The outcomes of these *ab initio* calculations are connected to the spin-Hamiltonian by an effective Hamiltonian method.^20,33^ Further, we use the *ab initio* results to calculate direction-dependent magnetizations in order to assess the magnetic anisotropy. The initial structures are obtained using density functional theory with the revTPSS^34^ and B3LYP^35^ functionals for gold and iron-doped gold clusters, respectively (see ESI† for further details on the theory, the resulting optimized geometries and spin densities). In all calculations, scalar relativistic effects have been taken into account by using the respective effective core potentials.

In the following section, the results are divided into two parts: (I) small Au_6_Fe and Au_7_Fe clusters, which represent even and odd electron count systems, are used to examine the methodology and critical calculation parameters. (II) Nano-sized truncated pyramidal Au_18_Fe and tetrahedral Au_19_Fe clusters, which represent realistic and thermodynamically stable models. Finally, we summarize the results and provide an outlook for future investigations.

## 2 Results & discussion

### 2.1 Au_6_Fe & Au_7_Fe – active space dependence

**Fig. 1:**
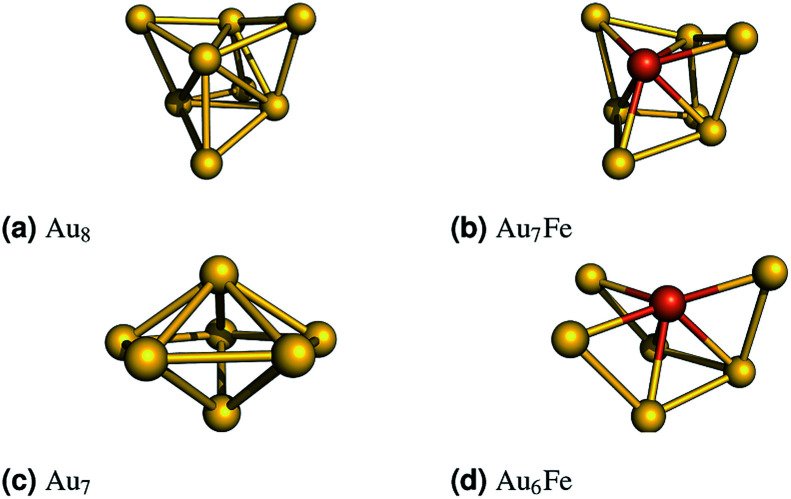
Left: The revTPSS/def2-TZVP optimized gold clusters are shown. The iron doped clusters were obtained by replacing one gold atom with an iron atom. The structures were optimized (B3LYP/def2-TZVP) for all reasonable spin states. The most stable (*S* = 2 for Au_6_Fe and *S* = 3/2 for Au_7_Fe) structures are shown on the right.

As mentioned in the Introduction, we begin by examining the central parameters of the CASSCF method, the size and composition of the active space, for two small test systems:

(1) Au_7_Fe, which represents clusters with an odd number of electrons, is derived from a three dimensional Au_8_ cluster (Fig. 1(a)). One gold atom was substituted and the resulting geometry was optimized for several spin states. The most stable (*S* = 3/2) geometry is shown in Fig. 1(b).

(2) Au_6_Fe, which represents clusters with an even number of electrons, is derived from a three dimensional Au_7_ cluster (Fig. 1(c)). Again, one gold atom was substituted and the resulting geometry was optimized for several spin states. The most stable (*S* = 2) geometry is shown in Fig. 1(d).

Using these optimized geometries, we calculated electronic ground and excited states for both systems with the SA-CASSCF/NEVPT2 method for several active spaces, which are denoted as CAS(*M*,*N*) where *M* is the number of electrons that are distributed over *N* spatial orbitals. For the even electron count system, Au_6_Fe, state averaging was done over 5, 45 states having a spin quantum number of *S* = 2, 1, respectively. These numbers represent the complete manifold of configuration state functions arising from 6 electrons in the five iron 3d orbitals. States with higher spins (*S* = 3) would require charge transfer type excitations, which are expected to have significantly higher energies. This was confirmed by a test calculation for CAS(8,7) (see below).

The situation is different for the odd electron count system, Au_7_Fe. Here an extra electron, originating from the additional gold atom, gives rise to three scenarios: (I) the electron stays in a delocalized 6s-type orbital interacting with the six 3d electrons. (II) The 6s electron is transferred into one of the localized 3d orbitals, resulting in seven 3d electrons. (III) A 3d electron is transferred from the iron into a delocalized 6s-type orbital, leaving five 3d electrons. It is expected that larger active spaces are needed to describe these scenarios appropriately. Further, from a computational and methodological point of view, not all possible states should be included in the calculation. Therefore, we only include 5, 20, and 20 states for *S* = 5/2, 3/2, and 1/2, respectively. Our decision to include only a subset of all possible excited states is justified by the following two considerations:

(1) The total number of possible states for an active space with 6 orbitals and 7 electrons (CAS(7,6)) is already 300.^36^ Due to the state averaging procedure, the ground state wavefunction is described less accurately. In addition, the multireference perturbative treatment of each state becomes rather cumbersome. Of course, this problem increases exponentially for larger active spaces.

(2) As shown by Atanasov *et al.*^20^ it can be expected that higher lying excited states contribute less to the zero-field splitting parameters, because of prefactors that include inverse excitation energies.

In addition, a second approach with seven electrons in only five d-orbitals has been tested. One assumes that the spin polarization arises only from the magnetic dopant. All possible states can be included in this case, however, the drawback of this approach is that only *S* = 3/2 and *S* = 1/2 states are possible. This not only excludes the possibility of a *S* = 5/2 ground state, but also neglects couplings between *S* = 3/2 and *S* = 5/2 states which might be important.

In Table 1, the first nonrelativistic excitation energy, *E*_ex_, the axial ZFS parameter, *D*, the rhombicity parameter, *E*/*D*, and the shifts of the **g**-tensor are listed for different calculation set-ups. Starting with Au_7_Fe (upper part of Table 1), five different active space compositions have been examined. For the smallest possible active space, (a)-CAS(7,6), which consists of seven electrons in six orbitals (five 3d orbitals and one 6s orbital), it was not possible to converge the calculation to a reasonable result. This minimum active space was therefore augmented with 2 and 4 Au 6s-type orbitals, leading to (b)-CAS(9,8) and (c)-CAS(11,10), respectively. *E*_ex_ is found to be similar for both calculations at around 2900 cm^−1^. Including more excited states ((d)-CAS(9,8)) or a second d-shell ((e)-CAS(7,11)) leads to a slightly reduced energy gap. Finally, also the results of the (f)-CAS(7,5) do not deviate significantly from all the other calculation setups. For all calculations, a ground state spin quantum number of *S* = 3/2 and a rather small absolute axial ZFS parameter is found. This, and the fact that all shifts of the **g**-tensor are quite close together, indicate a dominant isotropic magnetization behaviour. The values for the rhombicity parameter vary but, for such small |*D*| values, this is not surprising. We conclude that larger active spaces are necessary to appropriately describe the odd electron count system. The active space (c)-CAS(11,10) is sufficiently large while still computationally feasible and will therefore be used for further investigations. In addition, we will compare the results to calculations of the second “d-only” (f)-CAS(7,5) approach.

**Table 1:** The first nonrelativistic excitation energy (*E*_ex_), the axial ZFS parameter (*D*), the rhombicity parameter (*E*/*D*), and the shifts for the **g**-tensor are calculated with different the active space sizes in the SA-CASSCF/NEVPT2 approach. In the first column the active space CAS(*M*,*N*) is given, where *M* and *N* are the numbers of electrons and spatial orbitals, respectively. The second column labels the character of orbitals in the active space. The number of states included in the calculation is given in the third column. We use the following terminology: for a calculation with *N*(*S*_1_, ***S***_2_, *S*_3_) = [*N*_1_, *N*_2_, *N*_3_], *N*_*i*_ represents the number of included states with spin quantum number *S*_*i*_. The ground state spin quantum number (here *S*_2_) is indicated by boldface

	orbitals	# of states *N*({*S*_*i*_})	*E*_ex_ [cm^−1^]	*D* [cm^−1^]	*E*/*D*	*g*-Shifts [Δ*g*_*xx*_, Δ*g*_*yy*_, Δ*g*_*zz*_]
**Au**_**7**_ **Fe**						
(a)-CAS(7,6)	5x 3d, Au_s_	*N*(5/2, **3/2**, 1/2) = [5, 20, 20]	—	—	—	—
(b)-CAS(9,8)	Au_s_, 5x 3d, 2x Au_s_	*N*(5/2, **3/2**, 1/2) = [5, 20, 20]	2801.1	−2.39	0.089	[0.12, 0.12, 0.13]
(c)-CAS(11,10)	2x Au_s_, 5x 3d, 3x Au_s_	*N*(5/2, **3/2**, 1/2) = [5, 20, 20]	2947.0	1.36	0.147	[0.10, 0.12, 0.12]
(d)-CAS(9,8)	Au_s_, 5x 3d, 2x Au_s_	*N*(5/2, **3/2**, 1/2) = [5, 40, 40]	2611.5	−1.47	0.212	[0.12, 0.12, 0.14]
(e)-CAS(7,11)	5x 3d, 5x 4d, Au_s_	*N*(5/2, **3/2**, 1/2) = [5, 20, 20]	1656.7	−3.86	0.003	[0.35, 0.35, 0.45]
(f)-CAS(7,5)	3d	*N*(**3/2**, 1/2) = [10, 40]	1853.4	−1.66	0.005	[0.36, 0.36, 0.41]

**Au**_**6**_ **Fe**						
(a)-CAS(6,5)	5x 3d	*N*(**2**, 1) = [5, 45]	447.6	−45.11	0.030	[−0.02, 0.13, 0.93]
(b)-CAS(8,7)	Au_s_, 5x 3d, Au_s_	*N*(**2**, 1) = [5, 45]	183.0	−60.71	0.003	[−0.10, −0.01, 1.10]
(c)-CAS(10,9)	2x Au_s_, 5x 3d, 2x Au_s_	*N*(**2**, 1) = [5, 45]	413.7	−50.93	0.008	[−0.03, 0.04, 0.94]
(d)-CAS(12,11)	3x Au_s_, 5x 3d, 3x Au_s_	*N*(**2**, 1) = [5, 45]	391.0	−48.23	0.010	[−0.02, 0.04, 0.90]
(e)-CAS(10,9)	2x Au_s_, 5x 3d, 2x Au_s_	*N*(3, **2**, 1) = [1, 5, 45]	483.2	−44.99	0.039	[−0.02, 0.12, 0.89]
(f)-CAS(6,10)	5x 3d, 5x 4d	*N*(**2**, 1) = [5, 45]	395.5	−42.81	0.020	[−0.02, 0.11, 0.92]

Similar calculations can be done for the even electron count system, Au_6_Fe. The results are shown in the lower part of Table 1. Set-up (a)-CAS(6,5), represents the minimum active space size, consisting of five iron 3d orbitals with 6 electrons. For the set-ups (b) to (d), the active spaces are systematically expanded, by including one occupied and one unoccupied, delocalized valence orbital which both have dominant Au 6s character. For these set-ups, the ground state spin quantum number is *S* = 2, in accordance with the B3LYP calculations. The first excitation energies vary, but no trend can be found with respect to increasing active space size. Similarly, the axial ZFS parameter and the rhombicity parameter vary slightly as a function of the active space size. A higher multiplicity state was included in the fifth set-up, (e)-CAS(10,9), which, due to its high energy, has a negligible influence on the ZFS parameter. Finally, a second d-shell was included in the active space, resulting in set-up (f)-CAS(6,10). Again, only minor changes of a few wave numbers are observed. The qualitative result is similar for all tested set-ups: a large negative axial ZFS parameter in combination with a small rhombicity parameter, *E*/*D*, indicates a large axial magnetic anisotropy. This is supported by the shifts for the **g**-tensor, where one component (Δ*g*_*zz*_) is much larger than the other two. We conclude that, for the system with an even electron count, the smallest active space, (a)-CAS(6,5), captures all essential effects and is therefore used for further investigations.

The values shown in Table 1 demonstrate that the two systems exhibit very different properties. For example, for Au_6_Fe a high absolute axial ZFS parameter of 45 cm^−1^ with a negative sign and a very small rhombicity parameter of 0.03 is observed (for set-up (a)-CAS(6,5)). The resulting axial magnetic anisotropy is also reflected in the reported shifts for the *g*-values, where one shift is significantly larger than the other two (which are close to zero). The system is easily magnetized along one preferred orientation and, furthermore, it exhibits a high demagnetization barrier of |*D*|*S*^2^ = 180 cm^−1^. On the other hand, Au_7_Fe has a small axial ZFS parameter, which indicates more isotropic magnetization behaviour. This is supported by the shifts for the *g*-values, which are quite close together. Therefore, demagnetization is expected to occur significantly faster.

### 2.2 Au_6_Fe & Au_7_Fe – zero-field splitting & magnetizations

**Fig. 2:**
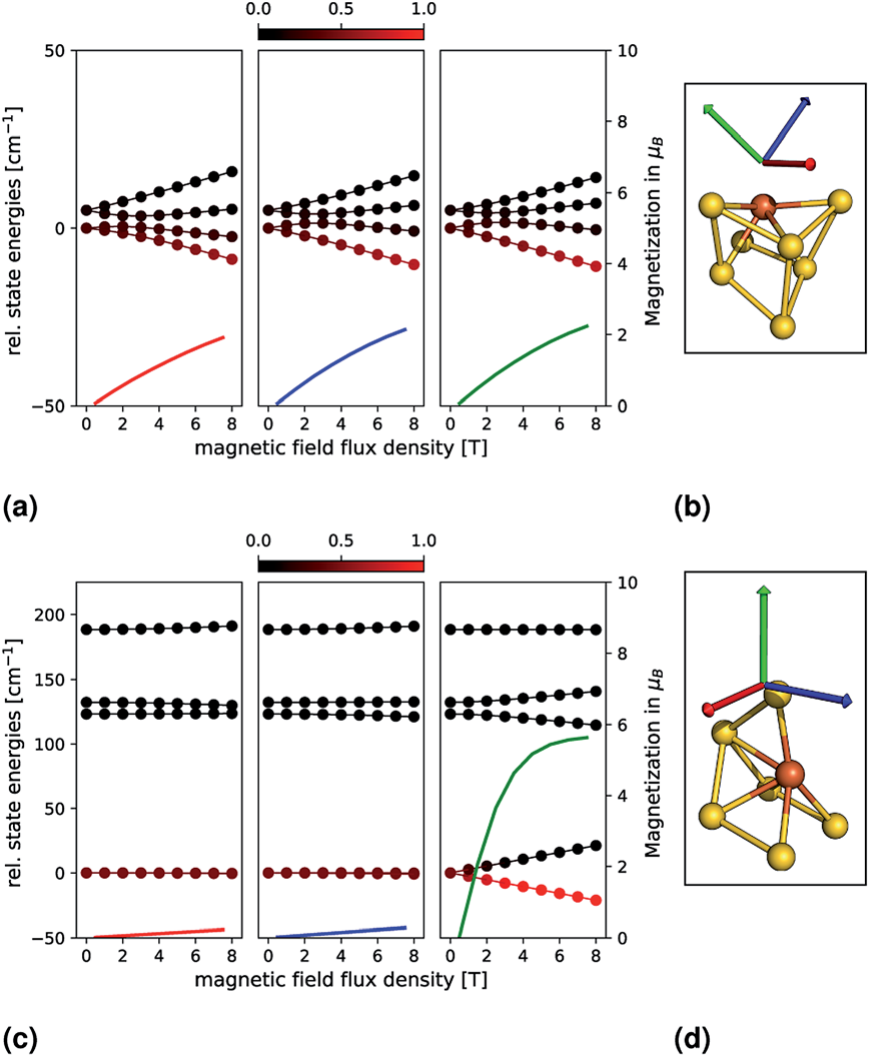
Relative state energies (solid lines with bullets) and magnetizations (solid lines) for the Au_7_Fe (top) and Au_6_Fe (bottom) are shown as a function of an external magnetic field. The principal axes of the **g**-tensor (calculated with the spin-Hamiltonian formalism) have been used as directions, which are indicated by coloured arrows on the right and correspond to one magnetization. Each energy data point is coloured by its Boltzmann population, from 0 (black) to 1 (red), at a temperature of 10 K.

In order to examine the properties of Au_6_Fe and Au_7_Fe in more detail, we explicitly simulate the influence of an external magnetic field. This is shown in the upper and lower parts of Fig. 2, which correspond to Au_7_Fe and Au_6_Fe, respectively. Each cluster, as well as three coloured arrows (each indicating one direction of the magnetic field) are shown in the insets (b) and (d). The dependence of the state energies and calculated magnetizations as a function of the external magnetic flux density is shown in insets (a) and (c). The colour of each magnetization graph corresponds to the colour of one arrow on the right to illustrate the direction of the external field. Each energy data point is coloured by its Boltzmann population, from 0 (black) to 1 (red), at a temperature of 10 K.

For Au_7_Fe, shown in the upper part of Fig. 2 (insets (a) and (b)), the *S* = 3/2 electronic ground state splits into two degenerate *M*_S_ = ±3/2 and *M*_S_ = ±1/2 magnetic states, independent of the value of *E*/*D* (Kramers theorem^20^). As expected from the small |*D*| value, the energetic separation is small. Further splitting is observed upon interaction with the external magnetic field. The three directions correspond to the principal axes of the **g**-tensor (*i.e.*, where the **g**-tensor is diagonal). The axes are then chosen such that their corresponding principal values fulfil g_*zz*_ > g_*yy*_ > g_*xx*_. The relative state energies vary with the magnetic field, and a magnetization is observed. As expected from the calculated shifts of the *g*-values and the small |*D*| value, an almost perfect isotropic magnetization behaviour is found.

For Au_6_Fe, shown in the lower part of Fig. 2 (insets (c) and (d)), the behaviour is quite different. As expected from the negative value of *D*, the magnetic ground states exhibit *M*_S_ = ±2 followed by *M*_S_ = ±1 and *M*_S_ = 0. The magnetic ground states (*M*_S_ = ±2) are almost perfectly degenerate, a small energetic gap can be observed for *M*_S_ = ±1, which is due to the small but non-negligible value of the rhombicity parameter, *E*/*D*. Switching on an external magnetic field leads to two different observations, which are dependent on the orientation of the field:

(1) For a magnetic field along a direction parallel to the main anisotropic axis, there is a strong splitting of the *M*_S_ = ±2 and *M*_S_ = ±1 states as shown on the right panel of inset (c) in Fig. 2. As a function of the external magnetic flux density, the formerly equally populated magnetic ground states exhibit increasingly different Boltzmann populations and the magnetization increases dramatically.

(2) For a magnetic field along a direction perpendicular to the main anisotropic axis, very different behaviour is expected and observed, as shown in the middle and left panels of inset (c) in Fig. 2. The relative energies depend much less on the external magnetic flux density. As such, the Boltzmann populations are essentially constant, and no significant magnetization is observed.

### 2.3 Au_18_Fe & Au_19_Fe – stabilities

**Fig. 3:**
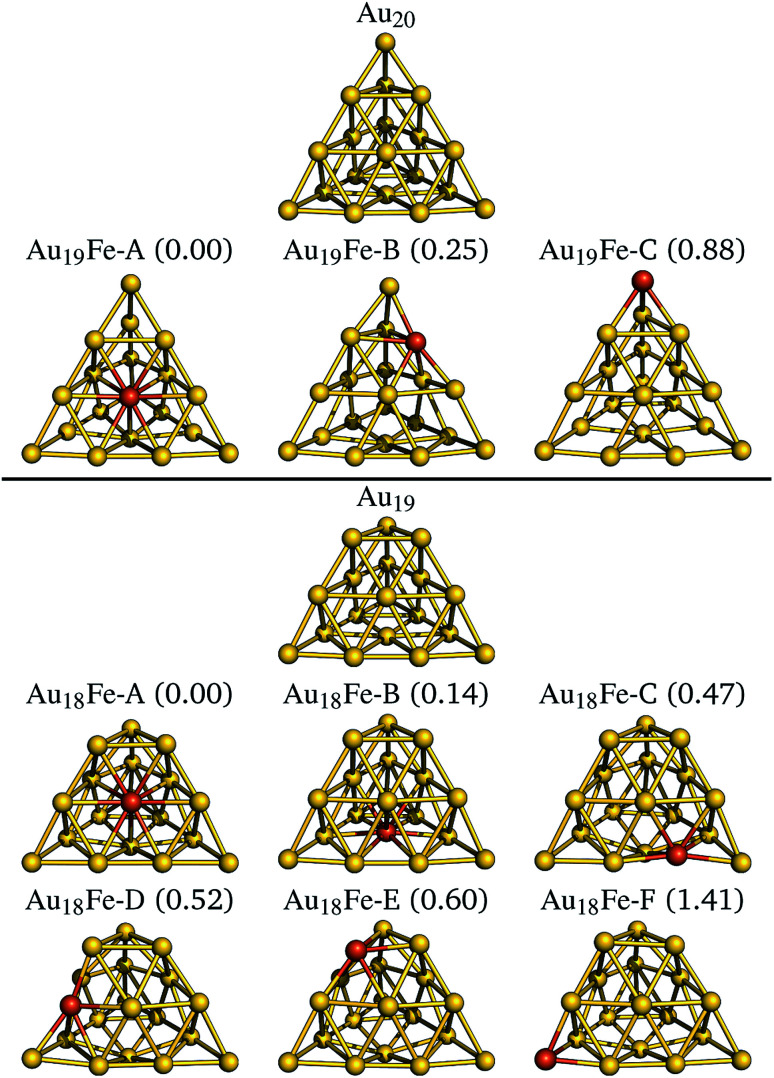
The revTPSS/def2-TZVP optimized, pyramidal Au_20_ structure is shown at the top. The derived and optimized iron doped clusters are shown below with increasing relative energies, given in brackets in units of eV. For all structures, the *S* = 3/2 spin quantum results in the most stable geometries. Below the truncated pyramidal Au_19_ and the six iron doped clusters are shown analogously. Here, the most stable clusters are found for the *S* = 2 spin quantum numbers.

We now examine two nano-sized iron doped gold clusters. The first class of systems is derived from the tetragonal Au_20_ cluster^37,38^ shown at the top of Fig. 3. The three optimized iron doped Au_19_Fe clusters are shown underneath in energetic order. The systems have odd electron counts and all ground states exhibit a spin quantum number of *S* = 3/2. The DFT derived relative energies are given in parenthesis in Fig. 3 and in Table 2. The face substituted system is most stable, followed by the edge (0.25 eV higher) and corner (0.64 eV higher) substitutions. The same result is found in the study of Tam *et al.*^12^ In Table 2, we further list the relative energies of the NEVPT2 calculations, using (c)-CAS(11,10), which support the energetic order of the B3LYP calculations. For most systems however, NEVPT2 predicts slightly higher relative energies.

**Table 2:** Relative energies, given in eV and calculated with two different electronic structure methods are listed for the three investigated Au_19_Fe and six Au_18_Fe clusters. The systems are labeled from A–C (Au_19_Fe) and A–F (Au_18_Fe) and correspond to the geometries shown in Fig. 3. For Au_18_Fe and Au_19_Fe, calculations have been done with (a)-CAS(6,5), (c)-CAS(11,10) and (f)-CAS(7,5), respectively. Further, the first nonrelativistic excitation energy (*E*_ex_), the axial and rhombicity parameter, and the shifts for the main values of the **g**-tensor are given

System	E(B3LYP) [eV]	E(SA-NEVPT2) [eV]	*E*_ex_ [cm^−1^]	*D* [cm^−1^]	*E*/*D*	*g*-Shift [Δ*g*_*xx*_, Δ*g*_*yy*_, Δ*g*_*zz*_]
**Au**_**19**_ **Fe – CAS(11,10)**						
Au_19_Fe-A	0.00	0.00	24.7	(9.69)	(0.050)	([−1.43, 0.02, 0.32])
Au_19_Fe-B	0.25	0.34	848.4	−9.52	0.197	[0.16, 0.18, 0.34]
Au_19_Fe-C	0.88	0.90	3848.9	3.37	0.138	[0.11, 0.17, 0.19]

**Au**_**19**_ **Fe – CAS(7,5)**						
Au_19_Fe-A	0.00	0.00	903.3	38.80	0.03	[0.12, 0.91, 0.94]
Au_19_Fe-B	0.25	0.25	1154.1	−20.47	0.31	[0.27, 0.47, 0.72]
Au_19_Fe-C	0.88	0.42	1839.1	11.31	0.04	[0.29, 0.47, 0.49]

**Au**_**18**_ **Fe – CAS(6,5)**						
Au_18_Fe-A	0.00	0.00	85.3	−29.52	0.200	[−0.25, 0.59, 0.96]
Au_18_Fe-B	0.14	0.06	111.1	19.34	0.107	[−0.28, 0.70, 0.85]
Au_18_Fe-C	0.47	0.76	458.2	−20.96	0.199	[0.04, 0.31, 0.66]
Au_18_Fe-D	0.52	0.76	508.6	19.49	0.179	[0.00, 0.42, 0.61]
Au_18_Fe-E	0.60	0.93	714.4	−35.25	0.011	[0.07, 0.08, 0.73]
Au_18_Fe-F	1.41	1.79	183.5	−51.91	0.015	[−0.00, 0.03, 1.08]

The second class of systems is derived from the truncated pyramid Au_19_ cluster^38^ shown at the lower part of Fig. 3. The six optimized iron doped Au_18_Fe clusters are shown underneath in energetic order. The systems have even electron counts and all ground states exhibit a spin quantum number of *S* = 2. The face substituted systems Au_18_Fe-A and Au_18_Fe-B are most stable, followed by the edge (Au_18_Fe-C and Au_18_Fe-D, 0.3 eV higher in energy) and corner (Au_18_Fe-E and Au_18_Fe-F, 0.1 eV higher in energy) substitutions. Quite surprising is the large energetic separation of 0.8 eV between the two corner substituted clusters, which is not observed for the two other substitution schemes (*i.e.* face and edge substitutions). Similar to the Au_19_Fe systems, the NEVPT2 calculations predict the same energetic order with slightly higher relative energies.

### 2.4 Au_18_Fe & Au_19_Fe – zero-field splittings and magnetizations

We now examine the magnetic properties of Au_18_Fe and Au_19_Fe. The first nonrelativistic excited state energy, the axial and rhombicity parameters, and the shifts of the main *g*-values for each system are listed in Table 2. The calculated values for the Au_19_Fe systems are all qualitatively different from each other. The face substituted system, Au_19_Fe-A, has a very small *E*_ex_ of only 24.7 cm^−1^ and the reported values are therefore given in parenthesis. Further, the ground state spin quantum number calculated with the NEVPT2 formalism for this cluster is *S* = 5/2, in contradiction to the B3LYP calculations. However, the gap between the ground *S* = 5/2 state and the lowest *S* = 3/2 state is quite small (only 63.3 cm^−1^). Calculations with basis set extrapolation and without state averaging might be needed in order to give a reliable prediction of the ground state spin quantum number but this is beyond the scope of the present paper. For the other two systems this problem does not occur. For the edge (Au_19_Fe-B) and corner (Au_19_Fe-C) substituted systems, we find first nonrelativistic excitation energies of 848 cm^−1^ and 3849 cm^−1^, respectively. Both systems exhibit a ground state spin quantum number of *S* = 3/2, in agreement with B3LYP. The axial ZFS and rhombicity parameters indicate a small rhombic anisotropy (*D* = −9.5 cm^−1^ & *E*/*D* = 0.197 for Au_19_Fe-B and *D* = 3.4 cm^−1^ & *E*/*D* = 0.138 for Au_19_Fe-C). This is supported by the shifts of main *g*-values reported in the last column of Table 2. For the edge substituted system, Au_19_Fe-B, all three values are close to each other (0.16, 0.18, 0.34), with one slightly larger than the other two. Even more isotropic values are found for the corner substituted system (0.11, 0.17, 0.19). It is worthwhile to compare the results to the second approach, *i.e.* where only d-orbitals have been considered. Most noteworthy, the qualitative trends are in agreement with the previously discussed ones. For example, the signs of the *D* values are consistent for both setups. However, the absolute values of *D* are slightly larger and the rhombicity parameters differ as well. For both setups, no indications of single molecule magnet properties are found for all three candidates.

The ground states of the even electron count systems, Au_18_Fe, all exhibit a spin quantum number of *S* = 2. Starting with the most stable face substituted systems, Au_18_Fe-A and Au_18_Fe-B, we find an axial ZFS parameter of −29.5 cm^−1^ and 19.3 cm^−1^, respectively. Both have a pronounced rhombicity parameter which is reflected in the tabulated shifts for the *g*-values. For both systems, all three values differ significantly (Table 2, last column on the right). The two edge substituted systems, Au_18_Fe-C and Au_18_Fe-D, which are approximately 0.3 eV higher in energy, exhibit magnetic properties similar to the face substituted systems although the values for *E*_ex_ are higher in energy. Again, a rhombic anisotropy is observed for both systems (the shifts for the *g*-values differ for each direction and a high rhombicity parameter *E*/*D* is found).

Very different magnetic properties are observed for the systems highest in energy, *i.e.*, the corner substituted iron doped gold clusters Au_18_Fe-E and Au_18_Fe-F. For both systems, a high absolute axial ZFS with negative sign is observed. Further, relatively small rhombicity parameters (0.011 & 0.015 for Au_18_Fe-E and Au_18_Fe-F, respectively) are found, which indicate strong axial magnetic anisotropies. The shifts for the *g*-values also indicate strong axial anisotropy: for both systems two *g*-value shifts are close to zero, while one is significantly larger.

**Fig. 4:**
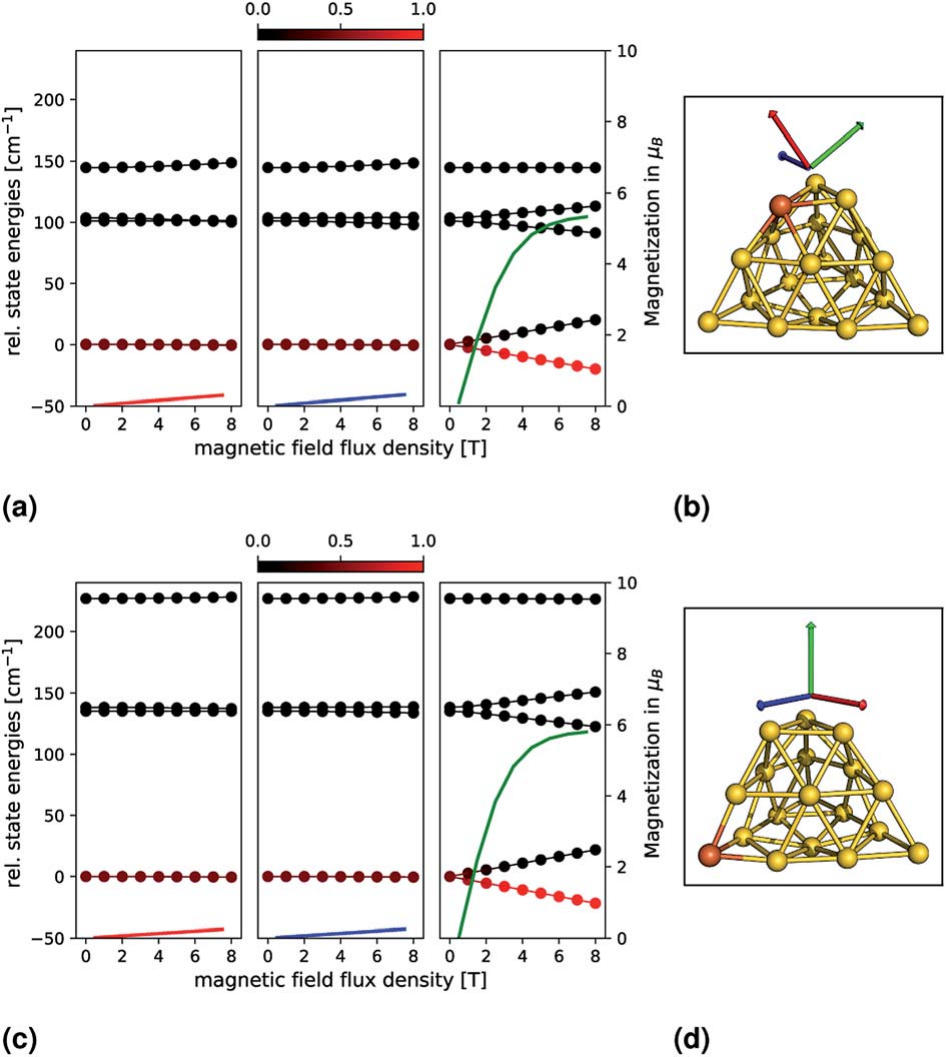
Relative state energies (solid lines with bullets) and magnetizations (solid lines) for the Au_18_Fe-E (top) and Au_18_Fe-F (down) clusters are shown as a function of an external magnetic field. The principal axes of the **g**-tensor (calculated with the spin-Hamiltonian formalism) have been used as directions, which are indicated by coloured arrows on the right and correspond to one magnetization. Each energy data point is coloured by its Boltzmann population, from 0 (black) to 1 (red), at a temperature of 10 K.

In order to analyze the magnetic anisotropy in more detail, we calculated magnetizations and relative state energies from the *ab initio* calculations (as previously for the small clusters). This is shown in Fig. 4 for the most promising candidates Au_18_Fe-E (top) and Au_18_Fe-F (bottom). The geometries and orientations (indicated by coloured arrows) of the external magnetic field are shown in (b) and (d). Insets (a) and (c) show, for each system, the relative state energies and magnetizations as a function of the external magnetic flux density. Again, note that the colour of one magnetization corresponds to the colour of one direction of the magnetic field. For Au_18_Fe-E, a ZFS in two *M*_S_ = ±2, two *M*_S_ = ±1 and one *M*_S_ = 0 is observed. For two directions along the principal axes of the **g**-tensor the state energies remain almost constant with respect to the external magnetic flux density. However, for one direction, the state energies vary drastically with increasing external magnetic flux density, as it can be seen in the right inset of panel (c). The lowest two states (*M*_S_ = ±2) vary most with the external flux density, followed by the third and fourth states (*M*_S_ = ±1). The fifth state (*M*_S_ = 0) shows no dependence. As a consequence, the ground state is dominantly populated and the magnetization increases dramatically. For the second candidate, Au_18_Fe-F, a similar behaviour is observed. However, the ZFS is much more pronounced compared to Au_18_Fe-E and the direction of main magnetization axis is oriented differently – almost perpendicular to the base of the truncated pyramid. For both systems, an impressive and almost perfect axial magnetic anisotropy is observed and we can estimate the demagnetization barrier to be |*D*|*S*^2^ = 141.2 cm^−1^ and 207.6 cm^−1^ for Au_18_Fe-E and Au_18_Fe-F, respectively. Both systems are therefore candidates for nanomagnets.

## 3 Summary

In conclusion, we find that the corner substituted systems Au_18_Fe-E and Au_18_Fe-F exhibit a very pronounced axial magnetic anisotropy. For both systems, a large absolute |*D*| with negative sign and an almost negligible rhombicity parameter *E*/*D* is found. The high axial anisotropy is further reflected in the shifts for the *g*-values and in the explicitly calculated relative state energies and magnetization (Fig. 4), where the axial anisotropy is demonstrated impressively. Thus our *ab initio* calculations indicate that both systems possess single nanoparticle magnetic properties and are therefore candidates for further (experimental) investigation. In future investigations, other (transition or f-shell) metal substitutions could be examined. Future studies could focus on the explicit time-dependent demagnetization dynamics. This would include the evaluation of transition rates between the different magnetic levels and a temperature-dependent propagation of the state populations until thermal equilibrium is achieved. Other interesting questions arise further from couplings of multiple spin centres (for example, Au_17_Fe_2_) or iron doped gold clusters bonded to ligands that could be used to tailor the electronic and magnetic properties of the system. A challenge is to identify thermodynamically stable clusters out of the huge geometrical space. Here, global optimization techniques like genetic algorithms^39^ could be very helpful.

## Conflicts of interest

There are no conflicts to declare.

## Supplementary Material

NA-001-C8NA00359A-s001
