# Supplementary material for: Iron doped gold cluster nanomagnets: ab initio determination of barriers for demagnetization
Source: Nanoscale Adv. 2019 Feb 12;1(4):1553–9. doi: 10.1039/c8na00359a (PMC9419490; doi:10.1039/c8na00359a)
Supplement: NA-001-C8NA00359A-s001 [file NA-001-C8NA00359A-s001.pdf]

Electronic Supplementary Material (ESI) for Nanoscale Advances.
This journal is © The Royal Society of Chemistry 2019

Iron Doped Gold Cluster Nanomagnets: *Ab Initio* Determination of Barriers for Demagnetization

Supporting Information

Christopher Ehlert[a] and Ian P. Hamilton[a]

Department of Chemistry and Biochemistry, Wilfrid Laurier University, 75 University Ave W, Waterloo ON N2L3C5, Canada

1 Methods

1.1 Geometries & Spin States

All calculations have been performed with the ORCA program[1]. Throughout our study, we used the def2-TZVP basis set[2] in combination with the resolution of the identity (RI) approximation and scalar relativistic effective core potentials. DFT was used to optimize geometries. For pure gold clusters, we used the revTPSS functional[3] (which performs well for gold clusters[4]). For the iron doped clusters, the B3LYP functional was used[5]. The initial structures were obtained by replacing an Au atom of the optimized gold cluster with a Fe atom, followed by optimization for low, medium and high spin states.

1.2 Calculation of Spin-Hamiltonian Parameters and Magnetizations

Spin-orbit and Zeeman effects are calculated within the quasi-degenerate perturbation theory (QDPT) formalism. A detailed description of the methodology is given by Atanasov et al.[6]. Therefore, we only summarize it briefly. The central task is the diagonalization of the matrix:

$$\langle \Psi_I^{\mathrm{SM_S}} \mid \hat{H}_{\mathrm{BO}} + \hat{H}_{\mathrm{SOMF}} + \hat{H}_{\mathrm{Z}} \mid \Psi_J^{\mathrm{S'M'_S}} \rangle = \delta_{\mathrm{IJ}}\delta_{\mathrm{SS'}}\delta_{\mathrm{M_SM'_S}} E_I^{\mathrm{S}} + \langle \Psi_I^{\mathrm{SM_S}} \mid \hat{H}_{\mathrm{SOMF}} + \hat{H}_{\mathrm{Z}} \mid \Psi_J^{\mathrm{S'M_S}'} \rangle \quad . \tag{1}$$

where $\hat{H}_{\mathrm{BO}}$ is the Born-Oppenheimer Hamiltonian, $\hat{H}_{\mathrm{SOMF}}$[7] is the spin-orbit mean field operator, and $\hat{H}_{\mathrm{Z}}$ is the Zeeman operator. $\hat{H}_{\mathrm{SOMF}}$ and $\hat{H}_{\mathrm{Z}}$ are diagonalized in the basis of nonrelativistic wave functions $\Psi_I^{\mathrm{SM_S}}$, where S and $\mathrm{M_S}$ are the spin and spin projection quantum number of the I-th state. $\hat{H}_{\mathrm{SOMF}}$ couples nonrelativistic wave functions with $\Delta S = \pm 1, 0$, which are obtained by a state averaged complete active space calculation (SA-CASSCF)[8]. The number of states that are included in the calculations, are given in the main text. During the state averaged orbital optimization process, the sets of states with different spin quantum numbers are weighted equally. The SA-CASSCF state energies are further corrected by the NEVPT2 method[9–12] and used in Eq. 1 (E_I^{S}).

We also calculate magnetizations, which can be defined with respect to a given Cartesian axis k as:

$$M_k = -\frac{\partial E}{\partial B_k} = \frac{\sum_n -\frac{\partial E_n}{\partial B_k} \exp\left(-\frac{E_n}{\mathrm{k_B}T}\right)}{\sum_n \exp\left(-\frac{E_n}{\mathrm{k_B}T}\right)} \qquad (\mathrm{k = x,y,z}) \tag{2}$$

where N_A and k_B are the Avogadro and Boltzmann constant, respectively. Here T is the temperature and E_n are energies of the magnetic sublevels, i.e., the eigenvalues of Eq. 1 for a given Zeeman operator. We obtain the partial derivatives of the energies by numerical differentiation of the state energies for an increasing external magnetic field flux density.

An effective Hamiltonian method is used to connect the *ab initio* results Eq. 1 with the Spin-Hamiltonian parameters[6,13]. One has to mention, that the system can only be described by this method, if the ground state is sufficiently separated from the first excited state. If this is not the case, additional terms are needed to describe the system[14]. Because this requirement is not always fulfilled, we calculate the first excitation energy, $E_{ex} = E_1 - E_0$ and, for cases with small values, we report the Spin-Hamiltonian parameters (which must be used with caution) in brackets.

To summarize, in order to calculate the Spin-Hamiltonian parameters and magnetizations of an iron doped gold cluster, the following steps must be taken:

1. Geometry optimizations, using B3LYP/def2-TZVP, for all reasonable spin quantum numbers.
2. For the most stable geometry found in step 1, a SA-CASSCF with subsequent NEVPT2 correction is performed.
3. The SA-CASSCF wave functions and NEVPT2 energies are used as ingredients for Eq. 1.
4. The effective Hamiltonian method is used to find the Spin-Hamiltonian parameters, i.e, the axial ZFS parameter D, the rhombicity parameter E/D and the three main values of the **g**-tensor g_{kk} (k=x,y,z).
5. For a certain orientation, we calculate the magnetization and relative state energies via Eq. 2 by using the eigenvalues of Eq. 1.

1.3 Sample Input File

```
!NEVPT2 def2-TZVP def2/JK
!moread
%moinp "qro.qro"

%casscf
  mult 5,3
  trafostep ri
  bweight 1
  nroots 5,45
  nel 6
  norb 5
  rel
    dosoc true
    gtensor true
    soctype 0
  end
end

* xyzfile 0 5 geopt.xyz
```

2 Results

2.1 Optimized Geometries

Element	X [Å]	Y [Å]	Z [Å]
Au_6Fe			
Fe	-0.26304	-0.80628	0.22421
Au	-0.88723	1.85232	-0.03842
Au	-2.91878	-0.34469	-0.37376
Au	-0.94171	0.15656	-2.22075
Au	1.19567	-1.57234	-1.72496
Au	-2.05672	-1.41329	1.99000
Au	-0.00407	0.76726	2.30381
Au_7Fe			
Fe	0.82063	0.82093	0.82077
Au	1.67141	-1.52717	1.67118
Au	-1.52728	1.67045	1.67066
Au	1.67097	1.67053	-1.52721
Au	-1.06212	-1.06184	1.09094
Au	1.09092	-1.06186	-1.06210
Au	-1.06192	1.09092	-1.06189
Au	-1.60261	-1.60197	-1.60236
$Au_{18}Fe - A$			
Au	-0.03769	4.71540	0.00511
Au	-0.05094	3.36970	-2.46453
Au	-1.52018	2.41060	-0.13194
Au	1.48580	2.42952	0.06743
Au	-0.10660	1.85120	-4.73367
Fe	-1.13391	0.76254	-2.37400
Au	1.70188	1.14596	-2.50308
Au	-2.82784	0.10129	-0.16720
Au	-0.01630	0.00297	0.33119
Au	2.79373	0.06280	-0.02074
Au	-1.48032	-0.55282	-4.74519
Au	1.33760	-0.61118	-4.76199
Au	-2.87957	-1.45379	-2.58785
Au	-0.03330	-1.94287	-2.60155
Au	2.83340	-1.46225	-2.60912
Au	-4.08084	-2.35533	-0.17528
Au	-1.36253	-2.51573	-0.11077
Au	1.32461	-2.51120	-0.15773
Au	4.04839	-2.37167	-0.25073
$Au_{18}Fe - B$			
Au	-0.00025	4.69669	0.00976
Au	-0.00020	3.39424	-2.48528
Au	-1.44064	2.36036	0.08018
Au	1.43676	2.35837	0.08072
Au	-0.00138	1.84224	-4.78580
Au	-1.62752	1.05163	-2.49710
Au	1.63066	1.05420	-2.49894
Au	-2.72379	0.03812	0.05163
Fe	-0.00045	-0.01913	-0.23569
Au	2.72228	0.03707	0.05015
Au	-1.40447	-0.62947	-4.82882
Au	1.40286	-0.62976	-4.82865

Au	-2.85046	-1.50450	-2.57810
Au	0.00090	-1.70540	-2.54050
Au	2.85209	-1.50237	-2.57957
Au	-4.02819	-2.37735	-0.14805
Au	-1.31702	-2.50492	-0.05410
Au	1.31684	-2.50736	-0.05374
Au	4.02738	-2.37750	-0.14977
$Au_{18}Fe - C$			
Au	-0.14009	4.62129	0.06673
Au	-0.04691	3.36034	-2.46724
Fe	-1.18628	2.22302	-0.20592
Au	1.527549	2.428751	-0.02935
Au	-0.04642	1.79677	-4.77027
Au	-1.87414	1.21418	-2.59547
Au	1.788976	1.159941	-2.55670
Au	-2.70583	0.12995	-0.07945
Au	-0.02354	0.00051	0.77133
Au	2.723199	0.033741	-0.04503
Au	-1.42674	-0.65156	-4.77243
Au	1.404294	-0.612680	-4.72871
Au	-2.88036	-1.44337	-2.55908
Au	-0.00972	-2.04515	-2.67954
Au	2.894658	-1.448070	-2.56374
Au	-4.04423	-2.33348	-0.16170
Au	-1.33469	-2.48379	-0.23328
Au	1.327668	-2.516057	-0.25521
Au	4.048026	-2.359209	-0.12657
$Au_{18}Fe - D$			
Au	-0.00030	4.70330	-0.28075
Fe	-0.00016	2.91648	-2.28008
Au	-1.47795	2.39766	-0.03987
Au	1.47768	2.39756	-0.03942
Au	-0.00065	1.91733	-4.66007
Au	-2.10115	1.29969	-2.61018
Au	2.10017	1.30018	-2.60994
Au	-2.72952	0.03411	-0.09421
Au	-0.00022	-0.00787	0.78126
Au	2.72896	0.03408	-0.09438
Au	-1.39427	-0.55212	-4.68426
Au	1.39352	-0.55185	-4.68397
Au	-2.86932	-1.50349	-2.58387
Au	-0.00017	-2.09699	-2.69888
Au	2.86874	-1.50289	-2.58404
Au	-4.05521	-2.36660	-0.17765
Au	-1.32388	-2.48894	-0.23624
Au	1.32416	-2.48866	-0.23664
Au	4.05494	-2.36582	-0.17843
$Au_{18}Fe - E$			
Au	3.86709	0.00021	-2.70849
Au	1.29823	0.00038	-3.88766
Au	1.96992	-1.46672	-1.36231
Au	1.96988	1.46689	-1.36205

Fe	-1.18569	0.00049	-4.54892
Au	-0.44452	-2.00752	-2.83450
Au	-0.44466	2.00789	-2.83384
Au	0.03946	-2.77396	-0.02861
Au	0.34943	-0.00017	0.53232
Au	0.03934	2.77361	-0.02791
Au	-3.18700	-1.38618	-3.62442
Au	-3.18717	1.38648	-3.62405
Au	-2.61169	-2.90508	-1.35252
Au	-3.22525	-0.00008	-1.07055
Au	-2.61194	2.90487	-1.35178
Au	-2.07730	-4.06450	1.15915
Au	-2.19780	-1.33739	1.16109
Au	-2.19807	1.33665	1.16155
Au	-2.07744	4.06369	1.16018
$Au_{18}Fe - F$			
Fe	-0.168758	-4.579937	-0.001348
Au	-2.461913	-3.372803	-0.001510
Au	0.005902	-2.418618	-1.472714
Au	0.004859	-2.419416	1.471287
Au	-4.770626	-1.847751	-0.002889
Au	-2.543238	-1.157258	-1.824603
Au	-2.545573	-1.159360	1.823550
Au	-0.077925	-0.062308	-2.814007
Au	0.468902	0.019446	0.000038
Au	-0.080490	-0.063800	2.813170
Au	-4.676066	0.621976	-1.424640
Au	-4.676935	0.621090	1.419385
Au	-2.553489	1.506759	-2.895630
Au	-2.642697	2.078603	-0.001157
Au	-2.556283	1.504944	2.893284
Au	-0.183860	2.407315	-4.070362
Au	-0.174658	2.498002	-1.340172
Au	-0.176196	2.497268	1.340393
Au	-0.187805	2.405270	4.070463
$Au_{19}Fe - A$			
Fe	-0.00001	1.23879	2.49018
Au	0.00189	-4.71630	0.38008
Au	-0.00016	-3.34281	2.76050
Au	-1.54257	-2.42316	0.30702
Au	1.54348	-2.42148	0.31081
Au	-0.00782	-1.80857	4.97930
Au	-1.70447	-0.97798	2.76790
Au	1.70383	-0.97820	2.77465
Au	-2.89820	-0.07731	0.28568
Au	0.00000	0.00000	0.00000
Au	2.89830	-0.07609	0.29263
Au	-0.00000	0.00000	7.06485
Au	-1.45815	0.91289	4.89513
Au	1.45884	0.91438	4.89592
Au	-2.78091	1.72137	2.70964
Au	2.77773	1.72392	2.70971

Au	-4.07565	2.42720	0.33433
Au	-1.33135	2.53447	0.31520
Au	1.32890	2.53599	0.31533
Au	4.07295	2.42948	0.33357
$Au_{19}Fe - B$			
Fe	-1.18291	0.67667	4.83455
Au	4.04600	2.45106	0.40539
Au	2.89441	-0.04013	0.25257
Au	1.26901	2.53101	0.27305
Au	2.81893	1.68462	2.75340
Au	1.59980	-2.40580	0.27588
Au	0.00000	0.00000	0.00000
Au	1.75289	-0.95595	2.75155
Au	-1.43352	2.49001	0.32796
Au	-0.03719	1.93968	2.80287
Au	1.59897	0.82429	5.01805
Au	0.06372	-4.69839	0.40706
Au	-1.49448	-2.40181	0.28967
Au	0.05153	-3.25521	2.73732
Au	-2.86982	-0.07452	0.40095
Au	-1.66204	-0.98910	2.81956
Au	0.10822	-1.77222	5.00377
Au	-4.16619	2.34262	0.64422
Au	-2.85862	1.59261	3.01107
Au	-0.00000	-0.00000	7.11280
$Au_{19}Fe - C$			
Fe	-0.00000	0.00000	7.06298
Au	-4.06392	2.41276	0.45733
Au	-2.88706	-0.06649	0.29532
Au	-2.83313	1.63207	2.81879
Au	-1.30461	2.51006	0.32113
Au	-1.56088	-2.41646	0.30167
Au	-1.68287	-0.96011	2.78732
Au	0.00000	0.00000	0.00000
Au	-1.48462	0.83858	5.06354
Au	0.03148	1.82192	2.79048
Au	1.40385	2.49756	0.33884
Au	-0.01825	-4.70367	0.43519
Au	-0.01357	-3.26367	2.79465
Au	1.49256	-2.40126	0.32751
Au	0.02648	-1.71007	5.04846
Au	1.62142	-0.92568	2.80351
Au	2.87946	-0.07647	0.39144
Au	1.51848	0.87364	5.11694
Au	2.88108	1.65404	2.89235
Au	4.16368	2.37568	0.56802

2.2 Spin Densities

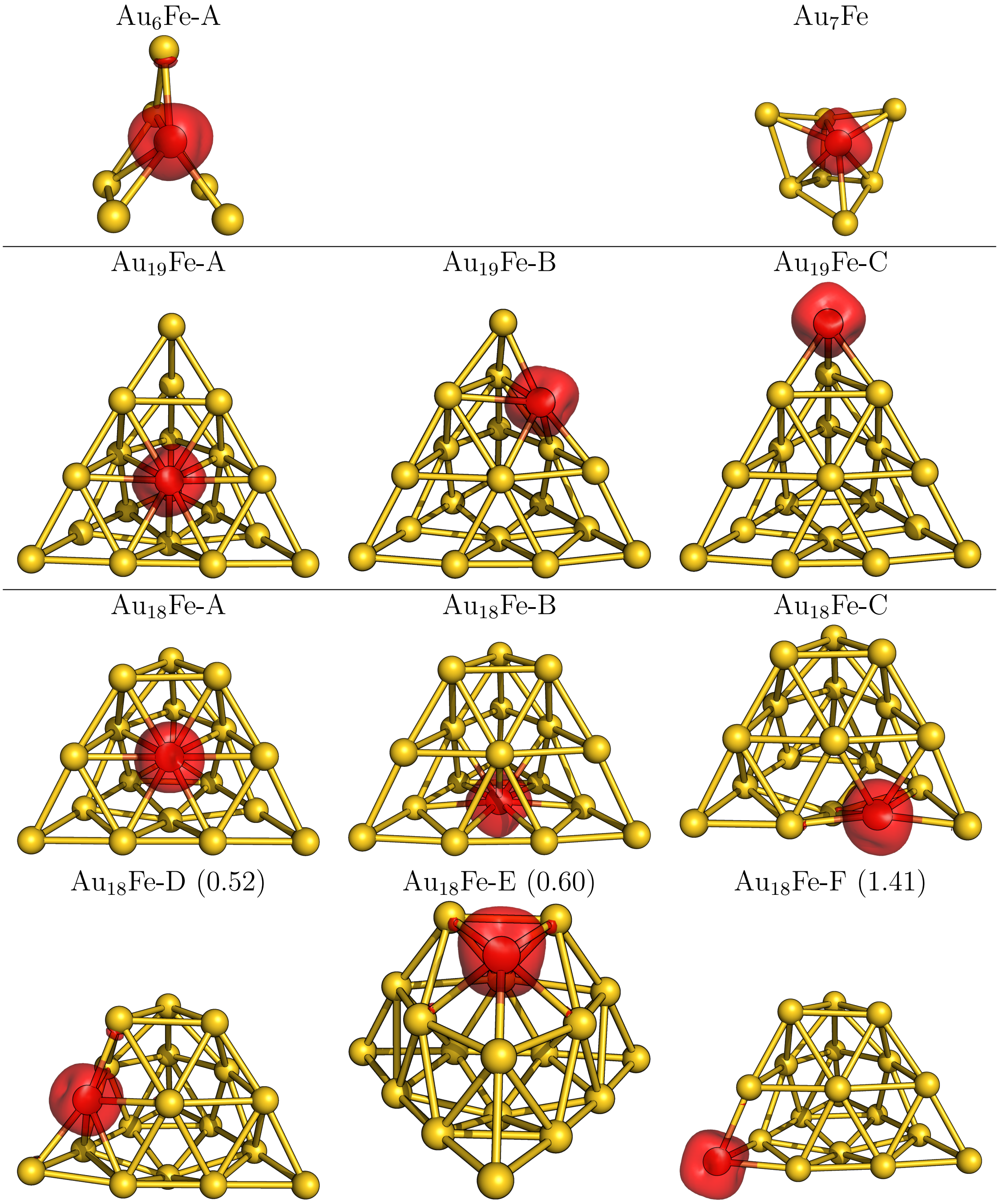

Figure 1: Spin densities for all investigated iron doped gold clusters based on B3LYP calculations. An isovalue of 0.01 has been used.

References

[1] F. Neese, *WIREs Comput Mol Sci*, 2017, **8**, e1327.

[2] F. Weigend and R. Ahlrichs, *Phys. Chem. Chem. Phys.*, 2005, **7**, 3297.

[3] J. P. Perdew, A. Ruzsinszky, G. I. Csonka, L. A. Constantin and J. Sun, *Phys. Rev. Lett.*, 2009, **103**, 026403.

[4] H. Baek, J. Moon and J. Kim, *J. Phys. Chem. A*, 2017, **121**, 2410–2419.

[5] A. D. Becke, *J. Chem. Phys.*, 1993, **98**, 5648–5652.

[6] M. Atanasov, D. Aravena, E. Suturina, E. Bill, D. Maganas and F. Neese, *Coord. Chem. Rev.*, 2015, **289**, 177–214.

[7] F. Neese, *J. Chem. Phys.*, 2005, **122**, 034107.

[8] P. Åke Malmqvist and B. O. Roos, *Chem. Phys. Lett.*, 1989, **155**, 189–194.

[9] C. Angeli, R. Cimiraglia, S. Evangelisti, T. Leininger and J.-P. Malrieu, *J. Chem. Phys.*, 2001, **114**, 10252–10264.

[10] C. Angeli, R. Cimiraglia and J.-P. Malrieu, *Chem. Phys. Lett.*, 2001, **350**, 297–305.

[11] C. Angeli, R. Cimiraglia and J.-P. Malrieu, *J. Chem. Phys.*, 2002, **117**, 9138–9153.

[12] C. Angeli, B. Bories, A. Cavallini and R. Cimiraglia, *J. Chem. Phys.*, 2006, **124**, 054108.

[13] R. Maurice, R. Bastardis, C. de Graaf, N. Suaud, T. Mallah and N. Guihéry, *J. Chem. Theory Comput.*, 2009, **5**, 2977–2984.

[14] M. Atanasov, D. Ganyushin, D. A. Pantazis, K. Sivalingam and F. Neese, *Inorg. Chem.*, 2011, **50**, 7460–7477.
